# Drugs for neglected tropical diseases: availability of age-appropriate oral formulations for young children

**DOI:** 10.1186/s13071-022-05546-7

**Published:** 2022-12-12

**Authors:** Issraa Al-Obaidi, Anna K. Krome, Karl G. Wagner, Kenneth Pfarr, Annette C. Kuesel, Hannah K. Batchelor

**Affiliations:** 1 Strathclyde Institute of Pharmacy and Biomedical Sciences, 161 Cathedral Street, Glasgow, G4 0RE UK; 2 Department of Pharmaceutical Technology and Biopharmaceutics, University of Bonn, 53121 Bonn, Germany; 3 Institute for Medical Microbiology, Immunology and Parasitology, University Hospital Bonn, Bonn, Germany; 4 German Center for Infection Research (DZIF), Partner Site Bonn-Cologne, Bonn, Germany; 5 UNICEF/UNDP/World Bank/WHO Special Programme for Research and Training in Tropical Diseases, World Health Organization, Geneva, Switzerland

**Keywords:** Neglected tropical diseases, NTD, WHO Roadmap, Oral formulation, Paediatric indications, Paediatric age-appropriate formulations, Preventive chemotherapy

## Abstract

It is recognised that paediatric indications and age-appropriate formulations are required to ensure that paediatric populations receive appropriate pharmacotherapeutic treatment. The lack of information on dosing, efficacy and safety data (labelling) is a well-recognised problem for all diseases affecting children. For neglected tropical diseases, the fact that they affect to a large extent poor and marginalised populations in low- and middle-income countries means that there is a low economic return on investment into paediatric development activities compared to other diseases [e.g. human immunodeficiency virus (HIV)]. This review provides an introduction to issues affecting the availability and development of paediatric population-relevant data and appropriate formulations of drugs for NTDs. We are summarising why age-appropriate formulations are important to ensure treatment efficacy, safety and effectiveness, outline initiatives to increase the number of paediatric indications/labelling and age-appropriate formulations, provide an overview of publicly available information on the formulations of oral drugs for NTDs relative to age appropriateness and give an introduction to options for age-appropriate formulations. The review completes with ‘case studies’ of recently developed paediatric formulations for NTDs, complemented by case studies for fixed-dose combinations for HIV infection in children since such formulations have not been developed for NTDs.

**Graphical Abstract:**

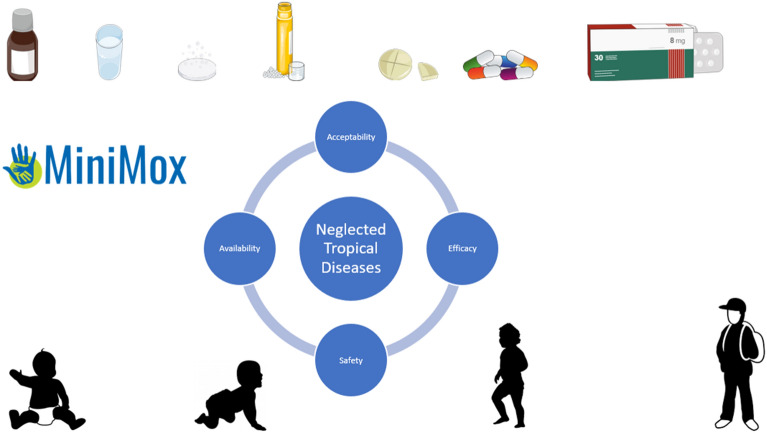

## Background

Neglected tropical diseases (NTDs) are a group of poverty-associated communicable diseases. The diseases that different organizations or authors consider NTDs differ slightly. The World Health Organization (WHO) regards 20 diseases/disease groups as NTDs. Most NTDs are parasitic diseases. Worldwide, NTDs affect more than one billion people. Considering the number of individuals affected, available diagnostics, local health care capacity and the efficacy and safety of available drugs, many NTDs are not addressed through management of individual cases, but via preventive chemotherapy (PCT), i.e. drug administration to specified populations irrespective of presence of symptoms or infection [1].

Due to limited or non-existing economic potential, few new drugs are being developed to treat NTDs [2]. Among 850 new therapeutics (drug products) registered in the USA and/or the European Union (EU) between 2000 and 2011, only five (0.59%) were indicated for NTDs. All were existing drugs repurposed for an NTD indication or a new formulation [3]. Repurposing, i.e. development of a new indication for an already approved drug, is much cheaper than development of a new drug (new chemical entity). This also applies for repurposing from veterinary to human use since regulatory requirements for non-clinical studies and manufacturing of drug (active pharmaceutical ingredient) and drug product (formulation) are very similar for veterinary and human drugs [4]. These 2000–2011 statistics show no improvement compared to 13/1393 (0.9%) approvals for NTDs from 1975–1999 [5]. Among 4006 phase 1 trials registered in ‘Pharmaprojects’ (a large commercial database of global pharmaceutical research and development) between 2000–2014, just 1.65% were for products intended for NTDs [6].

Several NTDs disproportionately affect children compared to adults [7]. As for most diseases affecting adults and children, the burden to children is compounded by lack of inclusion of paediatric populations in clinical trials (and thus missing information on age-appropriate dosing, efficacy and safety, i.e. paediatric indication and labelling) and/or lack of age-appropriate formulations [7]. Lack of paediatric labelling leads to ‘de-facto trials’ of the drug during off-label use for children with dosing based on the physician’s ‘best guess’, without all the safeguards that prospectively planned, comparative clinical trials approved by Ethics Committees and regulatory agencies provide, e.g. dose selection informed by thorough analysis of non-clinical and adult data, protocol-specified collection of safety and efficacy data and Ethics Committee-approved information to parents and, if applicable, children.

This review is designed to provide stakeholders with an introduction to issues relating to availability and development of paediatric population-relevant data and age-appropriate formulations of drugs for NTDs and to provide them with references for further information. We are (i) summarising why paediatric population-relevant data and age-appropriate formulations are important to ensure treatment efficacy, safety and effectiveness; (ii) outlining initiatives to increase the number of paediatric indications/labelling and age-appropriate formulations; (iii) providing an overview of currently available oral drugs for NTDs relative to age appropriateness and (iv) giving an introduction to options for age-appropriate formulations. We also provide ‘case studies’ of recent paediatric formulations for NTDs, complemented by case studies for fixed-dose combinations for HIV infection in children since such formulations have not yet been developed for NTDs. Table 1 provides an overview of formulation-relevant terminology.

**Table 1:** Short dictionary of drug formulation terminology

Term	Definition
Active pharmaceutical ingredient (API)	Any drug or drug substance used in a finished pharmaceutical product, whose use is to prevent, diagnose, treat or relieve symptoms of a disease or abnormal condition
Capsule sizes [8]	0: External diameter = 7.64 mm; length = 21.7 mm 1: External diameter = 6.91 mm; length = 19.4 mm 2: External diameter = 6.35 mm; length = 18.0 mm 3: External diameter = 5.82 mm; length = 15.9 mm 4: External diameter = 5.32 mm; length = 14.3 mm
Drug/drug substance	Any substance that is used to prevent, diagnose, treat or relieve symptoms of a disease or abnormal condition
Drug product/finished pharmaceutical product	A product that contains one or more APIs. Products can exist in many forms including tablets, capsules, liquids, creams and patches. They can also be administered in different ways, e.g. orally, as an injections into the vein, muscle or subcutaneous tissue or applied directly to the skin
Excipient	Substances other than the API that have been appropriately evaluated for safety and are intentionally included in a drug product/finished pharmaceutical product to serve different needs, e.g. stabilisation, enhancing solubility, filler
Solid oral dosage form	Refers to tablets or capsules or other solid dosage forms (made from powder) These can be sub-divided into those intended for immediate release and those intended to modify the release of the API
Tablets	Refers to: • Uncoated or coated (film-coated or sugar-coated) tablets that are intended to be swallowed whole • Unscored and scored* • Tablets that are intended to be chewed before being swallowed • Tablets that are intended to be dispersed or dissolved in water or another suitable liquid before being swallowed • Tablets that are intended to be crushed before being swallowed The term ‘tablet’ without qualification means an immediate-release tablet; any type of modified release version would have appropriate wording included in a qualifying description *Scored tablets may be divided for ease of swallowing, provided that dose is a whole number of tablets
Tablets (qualified)	Refers to a specific type of tablet: • Chewable—tablets that are intended to be chewed before being swallowed • Dispersible—tablets that are intended to be dispersed in water or another suitable liquid before being swallowed • Soluble—tablets that are intended to be dissolved in water or another suitable liquid before being swallowed • Crushable—tablets that are intended to be crushed before being swallowed • Scored—tablets bearing a break mark or marks where subdivision is intended to provide doses of less than one tablet • Orodispersible—tablets that are intended to disperse within the mouth, without the need for additional water, before being swallowed. These are also referred to as ‘melts’ as they ‘melt’ onto the tongue in the saliva • Sublingual—tablets that are intended to be placed beneath the tongue
Modified release tablets (qualified)	Refers to a specific type of modified release tablet including delayed-release tablets (gastro-resistant/enteric-coated tablets) and sustained-release tablets (extended-/prolonged-release tablets) • SR, CR, XR or ER is short for sustained, controlled or extended release, respectively, meaning that the tablet is formulated so that the API is released slowly over time • MR is short for modified-release, meaning that the tablet is formulated so that the API release is not instant but can be triggered by gastrointestinal conditions. It can also mean that the API is released slowly over time • Gastro-resistant or enteric-coated tablets are those where a coating is applied to prevent disintegration in the stomach so that the API will only be released once the tablet reaches the small intestine
Capsule	Refers to hard or soft capsules The term ‘capsule’ without qualification is *never* intended to allow any type of modified-release capsule
Capsules (qualified)	The term ‘capsule’ with qualification refers to gastro-resistant (such capsules may sometimes be described as enteric coated or as delayed release), prolonged release or another modified release form
Granules	Preparations that are issued to patient as granules to be swallowed without further preparation, to be chewed or to be taken in or with water or another suitable liquid Granules can be presented within capsules or sachets that are opened to extract the dose The term ‘granules’ without further qualification is *never* intended to allow any type of modified-release granules
Oral powder	Preparations that are issued to patient as powder (usually as single-dose) to be taken in or with water or another suitable liquid Powders can be provided within capsules or sachets that are opened to extract the dose
Oral liquid	Liquid preparations intended to be swallowed, i.e. oral solutions, suspensions, emulsions and oral drops, including those constituted from powders or granules, but not those preparations intended for oromucosal administration, e.g. gargles and mouthwashes Oral liquids presented as powders or granules may offer benefits in the form of better stability and lower transport costs. If more than one type of oral liquid is available on the same market (e.g. solution, suspension, granules for reconstitution), they may be interchanged and, in such cases, should be bioequivalent. It is preferable that oral liquids do not contain sugar and that solutions for children do not contain alcohol
Oral disintegrating/dissolving films or strips	Oral disintegrating/dissolving films or strips are defined as drug delivery systems that rapidly release API within the oral cavity by dissolving within the saliva present. The drug may then be absorbed directly from the mucosal surfaces of the oral cavity as well as swallowed and absorbed from the gastrointestinal tract

Source: [9, 10]

NTDs affect primarily low- and middle-income countries (LMIC). To ensure that stakeholders in LMIC can access at least the majority of references we provide, we reference only documents which are open access, available as author manuscripts in the US National Institutes of Health’s National Library of Medicine (PubMed Central, PMC, https://www.ncbi.nlm.nih.gov/pmc/), on the European Molecular Biology Laboratory European Bioinformatics Institute platform (Europe PMC, https://europepmc.org/), on institutional websites or accessible via HINARI. HINARI is the WHO initiative to provide free or very low cost online access to the major journals in biomedical and related social sciences to local, not-for-profit institutions in developing countries (https://partnership.who.int/hinari/about-us). Currently, institutions in 125 LMIC are eligible for HINARI (https://www.research4life.org/access/eligibility/). Publishers do not provide the same access to the publications in HINARI (https://portal.research4life.org/content/hinari; https://hinari.summon.serialssolutions.cm/#!/advanced) to all eligible LMICs. WHO itself has a relatively low level of access and we defined ‘accessible via HINARI’ as ‘accessible to the WHO staff co-author (ACK)’.

## The importance of age-appropriate oral formulations for drug efficacy and safety

Medicines can be administered via a variety of routes. Oral administration is preferred because of its familiarity and because products can be self-administered or administered by parents. Injections or infusions are usually expensive to procure, often require a temperature-controlled supply chain and need to be administered by a health care professional. This makes them not only unsuitable for PCT for NTDs but also suboptimal for patient treatment in resource limited settings.

Since 2007, WHO recommends liquid, granules or rapidly dispersible tablets for children to minimise the risk of choking [11]. A summary of proposed characteristics of pharmaceutical formulations for children and points to consider in pharmaceutical formulation of paediatric medicines were provided by WHO in 2012 [12, 13].

A summary of the main issues of using adult formulations is presented in Table 2.

**Table 2:** A summary of the main issues of using adult formulations in paediatric populations for NTDs

Main issue	Consequence
Lack of age-appropriate dose	Due to their smaller size children can require a lower dose yet the dose may not be readily available from the commercial adult product In addition, there may be limited clinical pharmacokinetic data upon which the paediatric dose is based
Lack of age-appropriate formulation	The nature of the adult product may make this unsuitable for use in children, for example tablets that are too large to be swallowed whole and are unsuitable to be crushed or split
Insufficient return on investment for development of a paediatric age-appropriate product	Due to the smaller population of paediatric patients and the economic situation in areas affected by NTDs there is limited scope for financial return on investment

### The impact of manipulation of age-inappropriate formulations to aid administration

Oral formulations available for NTD PCT for school-age and pre-school-age children do not necessarily have an age-appropriate size (Table 2). The mismatch between tablet sizes and the windpipe diameter of children [14] exposes them to the risk choking. There have been reports of 1–3% of children ≤ 36 months choking on tablets during deworming campaigns [15].

A review of swallowability of products for paediatric use revealed that the minimum diameter or lengths of tablets were not lower for drugs approved for use in children aged 2 to 5 years, 6 to 11 years or 12 to 18 years [16]. They were at the limit or beyond the children’s windpipe diameter for 6–11 and 2–5-year-old children [14], yet the age at which any product was labelled as having to be swallowed whole was 6 years. Interventions to teach children how to swallow pills were shown to be successful down to 2 years of age [17] but are unlikely to be feasible during PCT.

Lack of age-appropriate formulations forces health care providers to resort to ‘manipulation’ of the drugs to help administration, including crushing tablets, dispersing tablets in solvents or opening capsules and administering the contents of the capsule directly [11, 15, 18, 19].

Crushing or splitting of tablets not designed (i.e. scored) for splitting or use of the material within a capsule can result in particle losses and/or drug adhering to surfaces, resulting in the dose administered being lower than intended. Furthermore, any manipulation comes with a risk of contamination.

Tablet and capsule shapes are designed for easy swallowing through smooth surfaces and to minimise or eliminate contact of the API or excipients with the taste buds. A crushed or split tablet or the material taken out of a capsule will result in rough surfaces and sharp corners and may, whether dry or in water or food, take longer to swallow. The increased surface area will increase contact with the taste buds making administration uncomfortable or distasteful and result in poor acceptability. This was demonstrated for crushed and dissolved compared to whole tablets of levofloxacin (*n* = 3 and *n* = 9, respectively) and moxifloxacin (*n* = 10 and *n* = 4, respectively) administered to children able to self-report their experience (generally ≥ 5 years old): the majority disliked the taste, the look, the smell, the texture and/or the volume of the crushed and dissolved ‘formulation’ compared to a minority of those who took the whole tablets [20]. Fluoroquinolones are reported to have a bitter taste. Child-appropriate dispersible levofloxacin and dispersible moxifloxacin tablets prequalified by WHO in 2021 include taste masking (citrus fruit, peppermint, pineapple flavour) [21–23].

Importantly, for some types of formulations, ‘manipulation’ can affect drug absorption and thus exposure (the amount of API in the body over time), which determines both efficacy and safety. For example, tablets formulated using melt extrusion have to be swallowed whole, not broken up, crushed or chewed to ensure all active drug is absorbed. Administration of crushed 200/50 mg melt-extruded lopinavir/ritonavir tablets resulted in lopinavir and ritonavir exposure that was 45% and 47%, respectively, lower than after administration of whole tablets [24]. Drugs may be formulated in coated tables or capsules to protect them from degradation in the gastric environment or to protect the stomach from drugs that are local irritants. Crushing or administration of only the capsule content (dry or dissolved in liquid) removes the protective effect of the coating/capsule material putting children at risk of sub-therapeutic exposure or gastric irritation. Drugs may be formulated to include coatings or special excipients for release of the drug for absorption over time (prolonged/sustained release formulations) to enable once daily dosing. Crushing such tablets or administration of the dry or dissolved capsule content changes the product to an immediate release product, i.e. the full drug amount becomes available immediately for absorption (referred to as “dose dumping”) with the potential for toxic effects as well as subtherapeutic exposure.

### Acceptability of age-appropriate formulations

Availability of paediatric indications and age-appropriate formulations can increase the safety and efficacy of treatment but that depends on the patient’s adherence to the prescribed dosing regimen.

PCT for NTDs (or, for example, malaria chemoprevention [25, 26]) targets both morbidity and transmission reduction, adding an adherence dimension not encountered in patient treatment, i.e. ‘community adherence/compliance’. To achieve these PCT objectives, the whole population targeted, including those uninfected or infected but not experiencing ‘putatively adherence motivating’ symptoms, needs to ‘adhere’. A small percentage of infected individuals never taking the drug(s) can negatively impact transmission-related objectives [27]. A number of factors are known to impact compliance, but addressing them effectively in a local and culturally appropriate way remains a challenge [28–30].

Acceptability may significantly affect adherence. Acceptability has been defined as “the overall suitability of a formulation, including the dose volume or size and palatability” [20] and as “an overall ability and willingness of the patient and caregiver (defined as ‘user’) to use a medicinal product as intended (or authorized)” [31]. As of 2014, the European Medicines Agency (EMA) requires that acceptability of the proposed paediatric medicine is addressed in the ‘Paediatric Investigation Plan’ (PIP for further information on PIPs, see below). Acknowledging that “acceptability of and preference among the different paediatric dosage form(s) is known to vary between children”, with age, health status, behaviour, disabilities, background and culture being the most likely determining parameters, the EMA requires “dosage forms which facilitate the administration of a range of doses and that are acceptable to children of different ages” [32]**.**

The precise contribution of acceptability to adherence is difficult to establish. Oral formulation factors that may influence adherence include tablet size, shape and texture, tablet number, frequency of dosing, volume of liquid administered, palatability and requirement for administration with/without food.

Other factors impacting acceptability are packaging, container closure systems and written user’s instructions (product label and package leaflet) [32, 33], transport weight and volume (e.g. smaller tablets to aid swallowing or low liquid volumes to minimise any taste impact) [34] and logistical elements associated with preparation for administration (availability of food, clean water, juice and time needed).

## Initiatives to increase the number of paediatric indications and age-appropriate formulations

The need for paediatric indications and, if required based on the formulation developed for adults, age-appropriate formulations, has been recognised in the regulations of many countries and motivated initiatives globally.

### International Council for Harmonisation of Technical Requirements for Pharmaceuticals for Human Use

The ‘International Council for Harmonisation of Technical Requirements for Pharmaceuticals for Human Use’ (ICH, https://www.ich.org/, for members and observers, see https://www.ich.org/page/members-observers) issued guidance for paediatric trials in 2000 (E11), updated in 2017 (E11(R1)) [35]. E11 includes ‘pediatric extrapolation’ defined as “an approach to providing evidence in support of effective and safe use of drugs in the pediatric population when it can be assumed that the course of the disease and the expected response to a medicinal product would be sufficiently similar in the pediatric [target] and reference (adult or other pediatric) population”. Further consultation to reduce unnecessary paediatric trials and accelerate access to paediatric medicines is ongoing [36]. Furthermore, ICH has issued guidance on nonclinical safety testing in support of development of paediatric medicines (S11, [37]) and is working on guidance on bioequivalence studies for immediate-release solid oral dosage forms. The latter will complement guidance on establishing bioequivalence of different formulations, e.g. in the USA and the European Union (EU).

Widespread paediatric use of drugs approved for use in adults, i.e. off-label use, limits the return on investment sponsors (usually pharmaceutical companies) can expect for investment into paediatric indications and age-appropriate formulations. Consequently, regulatory agency mandates are needed to increase the number of paediatric indications and age-appropriate formulations.

### United States of America

The 1997 Food and Drug Administration Modernization Act resulted in the 1998 US Food and Drug Administration (US FDA) ‘Pediatric Rule’ [38], which required sponsors to submit proposed timelines for paediatric studies or information to support waivers or deferral. In 2002, the Best Pharmaceuticals for Children Act (BPCA) provided a 6-month extension of marketing exclusivity to sponsors submitting results of paediatric studies [39]. Following successful legal challenges to the ‘Pediatric Rule’, the 2003 ‘Pediatric Research Equity Act’ (PREA) [40] authorised the US FDA to require paediatric studies/age-appropriate formulations for certain drugs and biological products to ensure paediatric labelling. The 2012 Food and Drug Administration Safety and Innovation Act (FDASIA) finally obliged sponsors to identify in an ‘Initial Pediatric Study Plan’ (iPSP) highlighting the need for paediatric studies early during development of a new drug and to begin planning these studies [41]. In 2016, the US FDA reported that BPCA, PREA and FDASIA resulted in paediatric labelling of more than 600 products, including 149 since FDASIA as well as better international coordination of paediatric trials [42].

### European Union

The EU ‘Paediatric Regulation’ (EU-PR) [43], in force since 2007, mandates that for any new drug paediatric population needs are outlined early in development in a Paediatric Investigation Plan (PIP) and relevant studies are conducted prior to product authorization unless their requirement is deferred or waived by the European Medicines Agency (EMA) [44]. Furthermore, the EU-PR requires submission of data to inform addition of paediatric use information to the prescribing information for on-patent drugs. Considering the long time between initiation of development and regulatory authorization of new drugs, the EMA statistics on number of new paediatric products and paediatric indications authorised from 2004–2006 (29 and 12, respectively) and from 2012–2014 (30 and 38, respectively) provide a measure of the impact of the regulation [45]. The number of paediatric use related changes to prescribing information was 68 between 2004–2006 and 180 between 2012–2014. The EU-PR also introduced a ‘Paediatric-Use Marketing Authorisation’ (PUMA) for new paediatric indications with age-appropriate formulations of products which are off-patent or under a supplementary protection certificate. Drugs with PUMA benefit from 8-year data protection in parallel with 10-year market protection [46]. Despite PUMAs, various EU research framework programmes funding paediatric research on off-patent drugs and the EMA approving 20 PIPs for drugs potentially eligible for a PUMA, only six paediatric products had received a PUMA by 2018 [46]. The European Commission attributed the low PUMA number to factors impacting sponsor’s return on investment: widespread off-label use in children and unsatisfactory—from the pharmaceutical industry perspective—pricing agreements for products with PUMA which seem to reflect that European countries do not recognise the value of new paediatric indications or age-appropriate formulations for old (off-patent) drugs [46].

Through its European and Developing Countries Clinical Trials Partnership (EDCTP), the EU funds research for paediatric indications or formulations (https://www.edctp.org/projects-2/edctp2-projects/paediatric-drug-formulations-poverty-related-diseases-2019/) for primaquine (https://dpp-project.org/), moxidectin (https://www.minimox.eu/), HIV monoclonal antibodies (https://pedmab.w.uib.no/), acoziborole (https://dndi.org/global-networks/acozi-kids/) and new antiretroviral formulations for children in Africa (https://www.edctp.org/projects-2/edctp2-projects/paediatric-drug-formulations-poverty-related-diseases-2019/).

### WHO

In 2007, the World Health Assembly (WHA) passed the resolution “Better Medicines for Children” urging member states to ensure availability of age-appropriate dosage forms and strengths [47]. Thus, WHO launched, for example, the “Make medicines child size” initiative, developed the Model List of Essential Medicines for Children (EMLc) [10] and the WHO Model Formulary for Children [48], convened a global paediatric working group of representatives of National Medicines Regulatory Agencies to establish a ‘Paediatric Medicines Regulators’ Network’ [49] and provided points to consider during development of paediatric medicines [13]. Following the 2016 WHA resolution on paediatric medicines [50], WHO provided, for example, a toolkit for research and development of paediatric antiretroviral drugs and formulations [33], launched with partners the ‘Global Accelerator for Paediatric Formulations’ [51], reviewed innovative delivery systems for paediatric medicines [52] and provided procedures for paediatric drug optimisation [53].

### Other initiatives

Several partnerships are addressing the development of paediatric indications or formulations for selected NTDs or drugs. For example the “Drugs for Neglected Diseases initiative” (DNDi, https://www.dndi.org/) worked on paediatric benznidazole to treat Chagas disease in infants and children that was approved in 2017 for use in children aged 2–12 years with Chagas disease via the Accelerated Approval pathway. The Accelerated Approval pathway allows the US FDA to approve drugs for serious conditions where there is unmet medical need and adequate and well-controlled trials establish that the drug has an effect on a surrogate endpoint that is reasonably likely to predict a clinical benefit to patients [54]. DNDi is also working on a paediatric formulation of acoziborole to treat sleeping sickness (https://dndi.org/global-networks/acozi-kids/). The Medicines for Malaria Venture was involved in developing a child-suitable dispersible formulation of Artemether-Lumefantrine, launched in 2012 (https://www.mmv.org/access/products-projects/coartem-dispersible-artemether-lumefantrine/coartem-dispersible-facts). The TB Alliance has been involved in the development of age-appropriate dispersible formulations of the current standard tuberculosis (TB) treatment (isoniazid + rifampin + pyrazinamide + ethambutol) for children weighing > 5 kg (https://www.tballiance.org/portfolio/regimen/pediatric-hrze).

## Existing oral formulations for NTDs and their age appropriateness for paediatric populations

Some information on the formulations, limited presumably by proprietary interests, is made available by some regulatory authorities in the ‘Product Literature’ provided on publicly accessible data bases. These are listed in Table 3.

**Table 3:** Summary of sources used to extract information on age appropriate NTD formulations

Database	Weblink
‘Summary of Product Characteristics’ (SPCs) for medicines licensed for human use in the UK	https://www.medicines.org.uk/emc/
US FDA reviews and approved labels for products licensed for human use in the US	https://www.accessdata.fda.gov/scripts/cder/daf/index.cfm)
‘European public assessment reports’ (EPAR) for products approved by the EMA	https://www.ema.europa.eu/en/medicines/field_ema_web_categories%253Aname_field/Human
‘WHO Public Assessment Reports’ (WHO PAR) for drugs prequalified by WHO)	https://extranet.who.int/pqweb/medicines/prequalified-lists/finished-pharmaceutical-products
Drugbank: a web-enabled database containing comprehensive molecular information about drugs, their mechanisms, their interactions and their targets [55] Information on product shape, colour and size is also available	https://go.drugbank.com/
Product literature’ (labels/prescribing information) provided by the companies marketing the products on websites or as ‘package inserts’ have to be those approved by the relevant regulatory authority	

A summary of some of the aspects determining age-appropriateness of the oral formulations of drugs for NTDs, including the physical dimensions of solid oral dosage forms and any instructions for administration to children, is provided in Table 4.

**Table 4:** Summary of paediatric use-relevant characteristics of oral formulations used as per WHO NTD Roadmap 2021–2030 for treatment, control and elimination of NTDs [1]

Drug	Regulatory indication(s) [8th EMLc indication(s)]	Formulations(s)	Instructions for administration to children in regulatory label	References
Anthelminthics				
Albendazole	Lymphatic filariasis and the control of soil-transmitted helminthiasis (= 400 mg for children > 2 years; 200 mg for children aged 1–2 years) [Intestinal anthelminthic, antifilarial, cysticidal]	400 mg tablet scored oval tablet 19 mm x9mm x6mm 400 mg chewable tablet	The tablets should be taken with water on an empty stomach The tablets can be chewed or taken with water. Some people, particularly young children, may experience difficulties swallowing the tablets whole and should be encouraged to chew the tablets with a little water, alternatively the tablets may be crushed	[56] [57]
Mebendazole	Gastrointestinal infections caused by Ascaris lumbricoides (roundworm), Trichuris trichiura (whipworm), Necator americanus and Ancylostoma duodenale (hookworms) (500 mg for children ≥ 1 year) [Intestinal anthelminthic, cysticidal]	100 mg, 500 mg chewable tablet 500 mg scored chewable tablet: round, 16 mm diameter	Chew tablet completely before swallowing For subjects who have difficulty chewing the tablet, approximately 2 ml to 3 ml of drinking water can be added to a suitably sized spoon and the tablet placed into the water Within 2 min, the tablet absorbs water and turns into a soft mass with semi-solid consistency, which can then be swallowed	[58–60]
Antischistosomals and other antitrematode medicines				
Praziquantel	Preventive chemotherapy interventions for the control of schistosoma infections (600 mg for children ≥ 6 years) [Intestinal anthelminthic, antischistosomals and other antitrematode, cysticidal]	150 mg, 500 mg, 600 mg tablet 600 mg scored oval tablet 21 mm × 9 mm × 7 mm	Tablets should be swallowed whole with some liquid, preferably during or after meals	[61, 62]
Triclabendazole	Treatment of fascioliasis (10 mg/kg for children ≥ 6 years)[treatment of fascioliasis]	250 mg scored capsule shaped biconvex tablet	Swallow tablets whole or divide in half and take with water or crush and administer with applesauce	[63]
Antibiotics				
Azithromycin	Antibiotic for systemic use (10 mg/kg/day in infants > 1 year) [Watch group antibiotic]* [First choice: cholera; enteric fever; trachoma; yaws. Second choice: acute invasive bacterial diarrhoea /dysentery]	250 mg capsule. Capsules are not suitable for children < 45 kg Powder for oral suspension (when reconstituted with water, one bottle of POS contains 1200 mg in 30 ml (200 mg/5 ml)	Capsules should be taken at least 1 h before or 2 h after food	[64] [65]
Rifampicin	Tuberculosis (300 mg for children > 21–30 kg) [Antileprosy, antituberculosis]	150 mg capsule size 2, 17.6 mm × 6.39 mm 300 mg capsule size 0, 21.6 mm × 7.64 mm 20 mg/ml Oral liquid	Capsules should be taken on an empty stomach (at least 1 h prior to or 2 h after a meal) to ensure rapid and complete absorption	[61, 62]
Dapsone	Multibacillary leprosy Paucibacillary leprosy (100 mg for children ≥ 6 years) [Antileprosy]	25 mg, 50 mg, 100 mg tablet 100 mg scored round, 8 mm diameter	For oral administration	[66, 67]
Clofazimine	Tuberculosis (100 mg in adolescents 15 years or older weighing ≥ 30 kg) [Antileprosy, multidrug-resistant tuberculosis]	50 mg, 100 mg capsule 100 mg soft capsule	Should be taken with water and swallowed whole Should be taken with food to avoid stomach upset and improve absorption	[68]
Clarithromycin	Antibiotic for systemic use (7.5 mg/kg for infants > 6 months) [Watch group antibiotic]* [Second choice: Pharyngitis]	500 mg solid oral dosage form 500 mg film-coated oval tablet 18.5 mm × 8.1 mm 125 mg/5 ml, 250 mg/ml powder for oral liquid	The tablet should be swallowed whole with a sufficient amount of fluid (e.g. one glass of water) Clarithromycin film-coated tablets may be given irrespective of food intake	[69]
Moxifloxacin	Pneumonia, skin and abdominal (stomach area) infections (100 mg for infants > 5 kg) [Multidrug-resistant tuberculosis]	400 mg tablet 100 mg dispersible tablet, scored round 10 mm diameter (Micro Labs Ltd); caplet shape, scored 19 mm × 8 mm (Macleods Pharmaceuticals Ltd)	Dispersible tablet: Patients weighing ≥ 7 kg: The required number of tablets should be dispersed in approximately 10 ml of drinking water and the entire mixture should be swallowed. The mixture (tablets dispersed in water) should be used within 10 min. An additional volume of water should then be consumed immediately Patients weighing 5–6 kg: For administration of the correct dose, an oral syringe of 10 ml with 1 ml markings is needed. One tablet should be dispersed in exactly 10 ml of drinking water and mixed carefully; 8 ml of the mixture should be drawn up in the syringe and administered to the child	[23, 70, 71]
Antitrypanosomal				
Fexinidazole	First-stage (haemo-lymphatic) and second stage (meningo-encephalitic) of human African trypanosomiasis (600 mg for children ≥ 6 years and weighing ≥ 20 kg) [African trypanosomiasis]	600 mg tablet, round 13 mm diameter	Must be taken with food Swallow the tablets with a sufficient amount of fluid (e.g. one glass of water) Do not break or crush the tablets	[72]
Benznidazole	Chagas disease (5 mg/kg for children > 2 years) [American trypanosomiasis]	12.5 mg tablet round, 5 mm diameter; 100 mg tablet scored, round 10 mm diameter	May be taken with or without food Tablets may be made into slurry in a specified volume of water where the slurry must be drunk immediately following preparation and the cup used to prepare the slurry should be rinsed with additional water	[73]
Nifurtimox	Chagas disease (10 mg/kg in babies > 2.5 kg) [African trypanosomiasis, American trypanosomiasis]	30 mg round, scored tablet, 120 mg round, scored tablet	Must be taken with food Tablets can be made into a slurry as an alternative method of administration for patients who cannot swallow the tablet	[74]
Antiprotozoal drugs				
Miltefosine	Visceral leishmaniasis, cutaneous leishmaniasis (1.5 mg/kg for children ≥ 3 years) [Antileishmaniasis]	10 mg capsule size 3, 15.7 mm × 5.85 mm 50 mg capsule size 2, 17.6 mm × 6.39 mm	Administer with food to ameliorate gastrointestinal adverse reactions Swallow the capsule whole and do not chew it or break it apart	[75]
Antifungal drugs				
Itraconazole	Local and systemic candidiasis (3 mg/kg in infants > 1 month) [Chronic pulmonary aspergillosis, acute invasive aspergillosis, histoplasmosis, sporotrichosis, paracoccidiodomycosis, mycoses caused by T marneffei and chromoblastomycosis and prophylaxis of histoplasmosis and infections caused by T. marneffei in AIDS patients]	100 mg capsule size 0, 21.6 mm × 7.64 mm 10 mg/ml liquid	Must be taken immediately after a meal for maximal absorption Capsules must be swallowed whole with a small amount of water	[76]
Fluconazole	Local and systemic fungal infections: Mucosal candidiasis, invasive candidiasis, cryptococcal meningitis (6 mg/kg in infants > 1 month) [Antifungal]	50 mg capsule size 4, 14.3 mm × 5.33 mm 50 mg/5 ml oral liquid	The capsules should be swallowed whole and independent of food intake	[77]
Antifilarial drugs				
Ivermectin	Soil-transmitted helminthiasis, strongyloidiasis, onchocerciasis, lymphatic filariasis and scabies (3 mg in children > 15 kg) [Intestinal anthelminthic, Antifilarial, ectoparasitic infection]	3 mg scored tablet round, 6 mm diameter	The tablets should be taken with some water on an empty stomach. Do not eat any food within 2 h before or after taking this medicine In children < 6 years of age, the tablets should be crushed before swallowing	[78]
Diethylcarbamazine	Filarial infections due to *Wuchereria bancrofti*, Loa loa, *Brugia malayi* and *Brugia timori*, tropical eosinophilia, toxocariasis, 6—9 mg/kg daily in 3 divided doses [Antifilarial]	50 mg tablet scored tablet round 8 mm diameter 100 mg scored tablet round 12 mm diameter	Diethylcarbamazine should be administered after meals	[79]

^*^[Watch group antibiotic]; this is as an antibiotic recommended only for specific limited indications; for further information on categorisation of antibiotics by WHO the reader is directed to this web page: https://adoptaware.org/ (accessed July 2022)

Most of the available formulations are tablet/capsules. As outlined above, unless tablets are scored for splitting or other types of ‘manipulation’ before administration are explicitly stated in the ‘product literature’, the tablet/capsule should be swallowed whole since no data are available to support efficacy and safety after ‘manipulation’. This applies to 12/18 formulations (where size data were available) whose size ranges from 5 mm in diameter to 21 mm in length (Table 4). Only 2/18 products are designed as chewable/dispersible tablets and four can be crushed prior to administration.

## Requirements and options for age-appropriate oral formulations of drugs for NTDs

Paediatric formulations for NTDs should allow flexible dosage increments, have a minimal number of well characterised excipients, be palatable, safe and easy to administer and be stable regarding light. The environmental conditions in many areas where NTDs are prevalent also require products to be stable in high humidity and high temperature (ICH Zones III and IV) [80] since temperature-controlled storage and transportation are associated with high costs. Solid dosage forms such as tablets are typically more stable under these environmental conditions than liquids, semi-solids or suppositories.

Formulation design is not always simple. Each formulation needs to be designed based upon the drug that it contains, the patient population for whom it is designed and the context in which the drug will be administered. The physico-chemical properties of the drug can dictate the formulation strategy, e.g. a moisture-sensitive drug could not be formulated as an aqueous solution. The selection of excipients will depend upon the drug, the formulation design and the manufacturing process to ensure that the finished pharmaceutical product is of high quality and can meet regulatory agency quality, efficacy and safety requirements. Pharmaceutical excipients may be needed to mask bitter taste and/or to increase solubility, which may also affect the decision on which formulation is the most appropriate. The quantity of API, that is the dose, is also an important factor, since it determines the size and volume of the finished dosage form.

A short overview of options for oral drugs and considerations and data informing formulation choices is provided below with key advantages and disadvantages of different options summarised in Table 5.

**Table 5:** Key advantages and disadvantages of paediatric formulation options for oral solid dosage forms compared to conventional tablets, capsules or oral liquids

Chewable tablet	Dispersible tablet	Orodispersible tablet	Multiparticulate formulation	Oral films
Advantages				
• Lighter than liquids requiring less space during transportation	• Lighter than liquids requiring less space during transportation	• Lighter than liquids requiring less space during transportation	• Lighter than liquids requiring less space during transportation	• Lighter than liquids requiring less space during transportation
• Superior shelf-life to liquids	• Superior shelf-life to liquids	• Superior shelf-life to liquids	• Superior shelf-life to liquids	• Superior shelf-life to liquids
• Use similar excipients to conventional tablets, i.e. require no special manufacturing expertise	• Use similar excipients to conventional tablets, i.e. require no special manufacturing expertise	• Use similar excipients to conventional tablets, i.e. require no special manufacturing expertise	• Use similar excipients to conventional tablets, i.e. require no special manufacturing expertise	
• Can be used for large doses as tablet size is not limited by swallowability	• Can be used for large doses as tablet size is not limited by swallowability			
• Dosing is simpler than for liquid formulations or powders, which require doses to be measured before reconstitution		• Dosing is simpler than for liquid formulations or powders, which require doses to be measured before reconstitution		
	• Dose adjustment is possible following dispersion by taking a measured portion of the dispersed material		• Offer dose flexibility provided that there is a suitable device to accurately count the units required	• Offer dose flexibility provided that there is suitable markings/ scoring to allow accurate division
			• Coatings can be applied to provide modified release formulations	
			• Multiple drugs can easily be combined in a variety of ratios to provide flexible dosing in combination therapy	
**Disadvantages**				
• Potential palatability issues	• Potential palatability issues	• Potential palatability issues	• Potential palatability issues	• Potential palatability issues
• Often softer and therefore more fragile than conventional tablets and therefore need to be handled with care	• Often softer and therefore more fragile than conventional tablets and therefore need to be handled with care	• Often softer and therefore more fragile than conventional tablets and therefore need to be handled with care		• Often softer and therefore more fragile than conventional tablets and therefore need to be handled with care
	• They are hygroscopic and often need managed storage conditions or desiccants in packaging	• They are hygroscopic and often need managed storage conditions or desiccants in packaging		• They are hygroscopic and often need managed storage conditions or desiccants in packaging
• May require a clean water source (or alternative solvent) to facilitate swallowing	• Require a clean water source (or alternative solvent) to facilitate swallowing	• Require a clean water source (a typical tablet needs 5 to 10 ml of water to aid swallowing) and caregivers must spend time waiting for the tablet to dissolve	• Require a clean water source (or alternative solvent) to facilitate swallowing	• Require a clean water source (or alternative solvent) to facilitate swallowing
• Only suitable for children able to chew and cannot be used in the youngest population			• Need to be contained in a unit system (usually a capsule or sachet) which involves additional manufacturing steps	• Only suitable for potent drugs as the maximum loading (amount of drug/film) is low
• Insufficient chewing may still lead to a risk of choking or aspiration of large particles			• Additional steps in administration (opening the capsule/sachets) to transfer to the patient	• Require specialised manufacturing knowledge and facilities

Dispersion of tablets provides a mechanism to ensure that the tablets are easy to swallow by those unable to swallow the solid matrix. An orodispersible tablet is one that is designed to be dispersed within the oral cavity; thus, it is formulated to ensure very rapid dispersion to minimise any risks of choking plus to enable dispersion in the small volume of saliva that is present. A dispersible tablet is one that is designed to be pre-dispersed in a cup of water (or other solvent) or on a spoon with a few drops of solvent prior to administration. Often this pre-dispersion is described as forming a slurry. Orodispersible tablets minimise issues around administration as they are placed directly into the mouth whereas dispersible tablets require time and appropriate devices to prepare the dose, e.g. a dosing cup and availability of the solvent. The utensils used to prepare a dispersible tablet often need to be rinsed to ensure that the patient received the entire dose and that it is not stuck to the container. However, dispersible tablets offer the advantage of dose flexibility where the full dose can be dispersed, yet only a portion of the resulting slurry is administered to the patient.

## Case studies on age-appropriate oral formulations for NTDs and to treat HIV

The context and environmental conditions for which paediatric formulations for NTDs have been developed are presented in a series of case studies. This is complemented by case studies for fixed-dose combinations for HIV in children since such formulations have not been developed for NTDs and are used in the same endemic settings.

### Mebendazole strawberry-flavoured rapidly disintegrating chewable tablets

Mebendazole 100 mg and 500 mg chewable tablets are included in the eighth EMLc for treatment of intestinal helminths [10].

The formulations included in 2007 in the first EMLc were also chewable [81, 82] but too hard and too slowly disintegrating for small children to chew. This resulted in some children choking, spitting the tablets out or refusing to take the tablets, leading to WHO recommendations to crush the tablet for administration to small children [15].

A more rapidly disintegrating strawberry-flavoured chewable 500 mg tablet that converts to a semi-solid mass in less than a minute when mixed with water was developed to address these problems.

The new formulation has been included in the list of WHO prequalified medicinal products for mass treatment of children ≥ 1 year of age with gastrointestinal infections caused by *Ascaris lumbricoides*, *Trichuris trichiura* and hookworms (*Necator americanus* and *Ancylostoma duodenale*) [60, 83]. For children who have difficulty chewing the tablet, it can be dispersed in approximately 2 to 3 ml of drinking water on a suitably sized spoon. Within 2 min, the tablet absorbs water and turns into a soft mass with semi-solid consistency, which can then be swallowed [60, 84]. This makes the formulation both a chewable and a dispersible tablet.

The excipients used in the new formulation are all existing acceptable materials and the product would be suitable for manufacture using standard pharmaceutical equipment and processes. The inclusion of povidone and sucralose assists the rapid disintegration of the tablet [84]. The simplest and most economical process to produce tablets is direct compression where the excipients are mixed as a dry blend and subsequently compressed. Due to the poor flow characteristics of mebendazole powder, a direct compression process was not feasible and conventional fluid bed granulation for improved tabletability, screening, blending and compression processes were employed [60].

A chewable formulation was selected as WHO recommends chewable tablets for children < 5 years old; for those under 3 years of age, chewable tablets are to be crushed and administered with a small amount of water to reduce the choking hazard. Thus, this product would be suitable for children as young as 1 year of age [85]. The clinical testing of the product was initiated by conducting a relative bioavailability study of the new chewable formulation compared to the existing tablet in a healthy adult population which showed that systemic availability of the chewable formulation was higher than that of the existing product [86]. It is known that mebendazole has inherently poor systemic bioavailability, which is not important to its clinical effect as the intraluminal gastrointestinal concentration is of greatest importance for the efficacy of this drug [86].

A study to evaluate the safety and tolerability in children aged 2 to 10 years demonstrated a similar safety profile to the existing product [86]. In the study, 141/271 small (2–5 years) and 52/125 school-age (6 to10 years) children took the new formulation with water. For 29/271 small and 2/125 school-age children the tablet was broken up to facilitate chewing. Two children (age not specified) took the tablet after dispersal in water [86].

During a clinical study treating 278 children, including 141 children who were treated twice, with the new tablet, the tablet was dissolved in 2–3 ml water in a teaspoon for administration to small (1- < 3 years old) children (*n* = 24), while older children chewed the tablets. Among the small children, two gagged, one had difficulty swallowing and two spit out about half of the dose, but no child choked [87]. A more structured approach to comparing the acceptability for 3–12-year-old children of the new (*n* = 199 including seven ≤ 5-year-old children) vs. the old (*n* = 199 including ten ≤ 5 years old children) version of the chewable formulation included observation of the children while they took the drug and a questionnaire [88]. The old formulation was crushed and mixed with water for 3–5-year-old children while 6–12-year-old children were asked to swallow the tablet with a glass of water. Independent of age, children were encouraged to chew the new tablet and swallow it without water but were offered water afterwards. While the taste of the new formulation was liked by 175/184 (95%) children, 66/184 (36%) said they would not want to take it again compared to 47/181 (26%) of children who took the old formulation [88]. The investigators reported that many children appeared to have difficulties swallowing the chewed up new formulation with 87% accepting the offered water. They attributed this to swallowing becoming difficult because the chewed tablet absorbed the saliva and proposed that a glass of water should be given with the new formulation to resolve this problem [88].

A study in 2–4-year-old children used a validated data-driven approach [ClinSearch Acceptability Score Test (CAST)] to compare the acceptability of the new formulation with historical data for ‘hard tablets’. The authors concluded that the chewable tablet was “positively accepted” in children aged 2–4 years with acceptability decreasing with age. The percentage of children who took the drug after ‘alteration’ and with the help of an ‘extra device’ increased with decreasing age [89].

These data [86–89] suggest the new formulation is a great improvement, that small children should be given the new formulation dispersed in water (see Patient Information Leaflet (PIL) [83]) and be observed during and after taking the tablets to address potential issues (gagging, spitting out) and that older children need to be provided with water to drink while or after chewing the tablets.

### Praziquantel dispersible tablets

Praziquantel is included as 150 mg and 600 mg tablets in the WHO EMLc as an anthelminthic, as a 600 mg tablet as an ‘antischistosomals and other antitrematode medicines’ and as a 500 mg and 600 mg tablet in the ‘Complementary List’ for ‘Cysticidal medicines’ [90, 91]. Praziquantel is recommended by WHO for the treatment and prophylaxis of schistosomiasis in pre-school and school aged children [1, 90, 92].

Praziquantel was co-developed by Bayer and Merck in the 1970s (https://www.merckgroup.com/en/company/history/the-living-memory-of-merck/bilharziose.html, (accessed May 2022)). First approved by the FDA in 1982, it is currently approved for treatment of schistosomiasis, clonorchiasis and opisthorchiasis in children ≥ 1 year old [93]. The product is provided as a film-coated oblong tablet, has three scores and can thus be broken into four parts each with 150 mg of praziquantel [93]. The film-coated tablet contains the following excipients: corn starch, hypromellose, magnesium stearate, microcrystalline cellulose, polyethylene glycol, povidone, sodium lauryl sulphate and titanium dioxide [93]. Pharmacokinetic studies in adults demonstrated rapid and high (> 80%) absorption but also a low systematic bioavailability and considerable variations between individuals [94]. Generic oral tablets are available.

For children < 6 years of age, the tablet can be crushed or disintegrated and mixed with semi-solid food or liquid [93]. Due to the bitter taste, WHO recommends mixing with juice, syrup or honey for pre-school children [92]. The bitter taste was mainly attributed to the D-praziquantel [(*R*)-praziquantel] enantiomer of the racemic mixture [95], which does not contribute to efficacy but increases tablet size and may contribute to adverse effects [96, 97]. Research into an enantiomer-pure formulation was included as a priority in the UNICEF/UNDP/World Bank/WHO Special Programme for Research in Tropical Diseases (WHO/TDR) 2008–2013 programme and initiated with an ‘open science’ approach [98–100]. Development of a water-dispersible tablet was recommended by WHO in 2011 as a suitable age-appropriate formulation for praziquantel in preschool children [19]. The Pediatric Praziquantel Consortium (https://www.pediatricpraziquantelconsortium.org/), an international not-for-profit public–private partnership, was established in 2012 for the development, registration and provision of a praziquantel formulation suitable for pre-school children (https://www.pediatricpraziquantelconsortium.org/) [101]. Table 6 shows the target product profile [101].

**Table 6:** Target Product Profile for paediatric praziquantel formulations

Description	Praziquantel paediatric formulations using: (i) the racemic mixture of praziquantel (ii) only L-praziquantel
Indication	Treatment of schistosomiasis (*Schistosoma mansoni* and *S. haematobium*)
Target population	Children (3–6 months to 6 years) with proven schistosomiasis infection able to take oral medication and not receiving co-medication for other diseases
Dosage and administration	Orally disintegrating tablet (taste masked) administered orally (as intact tablet or dissolved in water) as a single-dose treatment (in mg/kg of body weight)
Stability in WHO zone IVb climatic conditions (hot, humid climate, 30 °C/75% RH)	Minimum case scenario: stable for 18–24 months Base case scenario: Stable for 24–36 months High case scenario: Stable for > 36 months
Packaging	Primary packaging: HDPE bottles with or without desiccant (low bulk, weight and volume packaging material) if feasible. Package sizes that Allow optimal use under public health programme conditions Approximately 50–100 units per bottle
Key statement	The new formulation will be suitable for paediatric use in Sub-Saharan Africa, Brazil and other endemic countries. It will be appropriate for use in both case management administration and community directed mass treatment (i.e. large-scale preventive chemotherapy). This will require further post-regulatory approval field studies to assess effectiveness

Source: [101]. For WHO climatic zone definitions see [102]

Two novel orodispersible tablet formulations were developed containing (i) the racemic mixture of praziquantel and (ii) only the biologically active L-praziquantel enantiomer. Ultimately the enantiomer-pure formulation was chosen. It contains mannitol and the sweetener sucralose [103], which enhance the palatability and reduce the bitter taste of praziquantel [104]. Mannitol is a useful excipient in orodispersible formulations, as it provides a pleasant mouthfeel, possesses high water solubility and is well tolerated [105]. Further excipients are: corn starch, magnesium stearate and colloidal anhydrous silica [103]. The new round 150 mg formulation has a diameter of 9 mm compared to the 600 mg scored 22 × 8 × 6 mm current tablet [101].

The clinical development programme to support the planned 2022 submission for a scientific opinion from the EMA for the enantiomer pure formulation (arpraziquantel) by Merck [106] included: two relative bioavailability studies [107], a randomised control phase 2 pharmacokinetic-pharmacodynamic (PK PD) dose finding study in *S. mansoni*- and *S. japonicum*-infected children and infants (clinicaltrials.gov identifier NCT02806263)[108], a palatability study [103] and a phase 3 efficacy and safety study in 3-month- to 6-year-old schistosoma-infected children, including a 4–6-year-old *S. mansoni*-infected cohort randomised to arpraziquantel or the currently available formulation (clinicaltrials.gov identifier NCT03845140) completed in November 2021 in Côte d’Ivoire and Kenya [106].

As per Article 58 of Regulation (EC) No. 726/2004 [109], the EMA provides a ‘Scientific Opinion’ for medicines not intended for marketing within the EU and eligible for ‘Article 58’, now referred to as ‘EU-M4all’ procedure [44]. Eligibility is assessed in collaboration with the WHO (https://www.ema.europa.eu/en/human-regulatory/marketing-authorisation/medicines-use-outside-european-union). For a ‘Scientific Opinion’ the EMA evaluates the medicines (including new formulations, pharmaceutical forms or routes of administrations) according to the same criteria applied for a EU marketing authorization. Medicines with a positive EMA Scientific Opinion (or approval by another stringent regulatory authority) are eligible for an abbreviated procedure for WHO Prequalification since no assessment of the underlying data is required and only data relating to the use of the product in the intended populations, settings or regions (notably stability data) may undergo assessment [110, 111]. Both approval by the EMA and WHO Prequalification can simplify the regulatory approval in endemic countries including through eligibility for the ‘Collaborative Procedures for Accelerated Registration’ (https://extranet.who.int/pqweb/medicines/collaborative-procedure-accelerated-registration.

The ‘Pediatric Praziquantel Consortium’ is working on establishing arpraziquantel production capacity in Brazil and Kenya [106].

### Nifurtimox-dispersible tablets

Nifurtimox is an antiparasitic compound included in the WHO EMLc for treatment of *Trypanosoma cruzi* infection (Chagas disease, American trypanosomiasis, 30 mg, 120 mg and 250 mg tablet) and as a 120-mg tablet in combination with eflornithine for treatment of second stage *T. brucei gambiense* human African trypanosomiasis [90].

Nifurtimox was first registered in the 1970s in Chagas disease-endemic countries in South America for treatment of Chagas disease. The original 120-mg tablet had one score line allowing division into two 60-mg doses. Dose adjustment is based on age and body weight [112], which was not appropriately supported by this formulation for paediatric patients.

In 2020, nifurtimox dispersible tablets, available in 30 mg and 120 mg dosage strength, were approved by the US FDA for children aged < 18 years, including new-borns weighing at least 2.5 kg [74].

The dispersible tablets have a functional score line so that they can be halved prior to administration. They can be swallowed whole and they can also be dispersed in 2.5 ml water on a medicine spoon prior to administration [74]. They are not designed for orodispersion (dispersion within the oral cavity). Once the product is dispersed it should be administered straight away with food [74].

The relative bioavailability of the intact and dispersed tablets has been measured as well as the impact of administration with food [112]. There was a slight difference in the bioavailability of the slurry where the overall exposure and peak concentration were reduced compared to the intact tablet [112]. However, food improved the overall exposure substantially resulting in the recommendation that nifurtimox should be administered under fed conditions [112].

Patient compliance with the new formulation was measured via pill count and a daily diary [113]. The pill counts revealed that compliance was > 90%, which suggests good acceptance of the formulation; no details on the diary were included in the literature report [113]. No data on assessment of the acceptability, taste or palatability of this new formulation of nifurtimox were identified.

The dispersible tablets are supplied as a bulk pack in a high-density polyethylene (HDPE) bottle with a 3-g desiccant capsule [114]. They need to be stored between 20 °C and 25 °C with excursions permitted to 15 °C and 30 °C, which may cause issues in some areas where American and African trypanosomiasis are prevalent (although in July 2022 there are no publicly available reports regarding stability to date). The formulation contains disintegrants (calcium hydrogen phosphate and maize starch) to aid in disintegration to form the slurry. The formulation also contained a surfactant (sodium lauryl sulphate) to aid in the dispersibility of the drug [114]. There is no sweetener in the dispersible formulation which may affect the acceptability of the product to patients.

### Multiparticulate formulations: Lopinavir-ritonavir products

Lopinavir + ritonavir is included in the EMLc as 40 mg + 10 mg combination products (capsule with oral pellets, added in 2017 for treatment of children 3 months to 3 years [115], oral granules in sachet added in 2019 [116] and a heat-stable 100 mg + 25 mg tablet for treatment and prevention (prevention of mother-to-child transmission and post-exposure prophylaxis) of HIV. The oral liquid formulation was removed from the EMLc as well as the EML in 2021 [90].

The oral liquid (400 mg + 100 mg/5 ml) formulation is not anymore included in the 22nd EML and the 8th EMLc [90]. This syrup formulation contained approximately 42% (v/v) ethanol and approximately 15% (w/v) proplylene glycol and needed to be stored at 2 ℃ to 8 ℃ [117]. The high alcohol content, combined with a very unpleasant taste, made it an unsuitable option for children and the requirement for administration with food further limited use in resource-poor settings, as did the cold supply chain requirement [24]. Toddlers often refused to take it or vomited afterwards [118].

Film-coated lopinavir/ritonavir tablets are available. They were formulated using melt extrusion. Such formulations have to be swallowed whole and not broken, crushed or chewed. Because crushing of tablets is common practice for administration to small children, a study comparing lopinavir and ritonavir exposure in 6–17-year-old children administered crushed or whole 200 mg/50 mg lopinavir/ritonavir tablets was conducted: lopinavir and ritonavir exposure was 45% and 47% lower, respectively, after administration of crushed compared to whole tablets [24]. Tablets (100 mg/25 mg) suitable for swallowing whole by children ≥ 10 kg are available [119].

#### Lopinavir-ritonavir granules for oral suspension

In October 2020, lopinavir/ritonavir 40 mg/10 mg granules for oral suspension were included in the list of WHO prequalified medicinal products for treatment of children 14 days and older weighing > 3 kg in combination with other antiretroviral agents [120]. This product can be mixed with water or soft foods (e.g. applesauce or porridge) to aid in administration. However, the granules should not be chewed or crushed. The granules contain the following excipients: copovidone, sorbitan monolaurate, colloidal silicon dioxide, ethyl cellulose, mannitol, acesulfame potassium, sodium stearyl fumarate and vanilla flavour [120]. The granules are packaged into a sachet to contain each unit dose.

#### Lopinavir-ritonavir oral pellets

An alternative formulation is lopinavir/ritonavir oral pellets. The pellets contain the following excipients: colloidal silicon dioxide, copovidone, hydroxy propyl methyl cellulose, polyethylene glycol, sodium stearyl fumarate, sorbitan monolaurate and talc. The pellets are provided within a capsule (size 1; 19.4 × 6.96 mm) which can be opened and the pellets sprinkled onto soft food to ease administration. The pellets must not be stirred, crushed, dissolved/dispersed in food or chewed and the entire dose should be administered immediately following mixing with soft food [121].

A New Drug Application was submitted to the US FDA in 2013 and the US FDA informed the company on 21 May 2015 of tentative approval including the information that final approval cannot be provided until after the end of the patent and exclusivity protection period of the reference product [122]. Final approval is not reflected on the US FDA website of FDA-approved drugs [123]. In June 2019, lopinavir/ritonavir 40/10 mg oral pellets were registered with the South African Health Products Regulatory Authority (https://www.sahpra.org.za/registered-health-products/ registration numbers 51/20.2.8/0123, 51/20.2.8/0124.123). Supply planning by WHO, the Interagency Task Team on Prevention of HIV Infection in Pregnant Women, Mothers and their Children and UNICEF began in 2015 [124]. Oral pellets are included in the 2021 Optimal Formulary and Limited-Use List for antiretroviral drugs for children [125].

The acceptability of this pellet formulation was evaluated within a two-period (4 weeks each) crossover relative bioavailability trial comparing syrup and pellets in HIV-infected infants (3 to < 12 months, *n* = 19) and young children (1 to < 4 years, *n* = 26) and tablets and pellets in older children (4 to < 13 years, *n* = 32) in two clinics in Uganda [126]. Caregivers selected the formulation for treatment from 8 to 48 weeks, answered standardised questionnaires at enrolment and 4, 8, 12 and 48 weeks after treatment start and provided comments at each visit. Pellet preference of caregivers of infants and young children, respectively, increased from enrolment (37% and 12%) to week 12 (72% and 64%) and decreased to week 48 (44% and 36%); for older children it decreased continuously from 41% via 19% to 13% [126]. Caregiver-reported issues with pellets were their bitter taste requiring taste masking food (honey). The role of bias by health care workers and training for supporting caregivers needs further investigation [126].

### Multiparticulate formulation: Lopinavir, ritonavir, abacavir and lamivudine combination

HIV medicines often combine multiple drugs into a single combination product, which can result in large tablets difficult to swallow for paediatric patients. A multiparticulate fixed dose combination (Quadrimune) of abacavir (30 mg), lamivudine (15 mg), lopinavir (40 mg) and ritonavir (10 mg) has been developed in partnership between a pharmaceutical company, Cipla Ltd, India, and the not-for-profit DNDi.

This ‘4-in-1’ formulation consists of granules (0.2–0.5 mm in diameter) that are filled into a capsule (capsule size is not reported) [127], which can be opened and the granules sprinkled onto soft food or into water or milk to aid administration and ease the swallowing process. This formulation does not require refrigeration, which makes it superior to the existing liquid products for HIV. The formulation includes a strawberry flavour to improve palatability for children.

The Lolipop Study (clinicaltrials.gov identifier NCT03836833) was a phase 1/2, open label, randomised crossover pharmacokinetic, safety and acceptability study of Quadrimune in HIV-infected children (> 4 weeks old and weighing ≥ 3 and < 25 kg at the time of enrolment). Within this study the acceptability of the formulation was investigated after at least 21 days of dosing as a description of factors that affect acceptability of the formulation as reported by the caregivers. Of the 31 interviewed caregivers, 30 (97%) reported administering the ‘4-in-1’ as 'very easy' or 'easy', and 22 (71%) reported that the child had no difficulty in swallowing it [128].

The PETITE study is an ongoing phase 1/2, open-label, single-arm study in term neonates who will receive a single dose of the ‘4-in-1’ formulation, followed by intensive pharmacokinetic sampling and safety assessments [129]. None of the 18 neonates included in the first cohort had difficulty swallowing the 4-in-1 formulation. Across 24 administrations, the capsule contents were administered with breast milk 17 times and with formula milk seven times. Swallowing of the ‘4-in-1’ when mixed with milk was reported to be easy by all caregivers for all 24 administrations. No neonate refused or vomited the ‘4-in-1’. Lopinavir/ritonavir exposures were extremely low, preventing its use in neonates. The low exposure may be at least partly due to the formulation using the excipient Eudragit E-PO to taste-mask both lopinavir and ritonavir. Eudragit E-PO is a polymer which is insoluble at pH > 5. The acidity in the stomach of neonates may not have been sufficient to ensure drug release.

## Conclusions

The solid oral drug products available for NTDs are primarily tablets and capsules that should be swallowed whole. The sizes of these products are all > 5 mm in diameter and many are much larger: the range was 5–21 mm for the longest dimension of the tablet with a mean value of 14 mm, and 10 of the 18 paediatric oral formulations listed in the Roadmap identified in Table 3 were > 10 mm based on their longest dimension. These large units are not suitable for younger paediatric populations because of swallowing issues and the risk of choking. Of the 18 solid oral drug products, six have information in the product label that indicates that data are available to support efficacy and safety when the product is chewed, crushed or split immediately prior to administration, making them appropriate for use in children. The recent development of child-appropriate formulations of mebendazole, praziquantel and nifurtimox as well as products for children with HIV through private-private or public–private partnerships is encouraging. Establishment of a mechanism for sharing lessons learnt beyond those that can be conveyed in publications could support development of paediatric formulations for NTD, ideally as per a ‘priority list’ established by all stakeholders. The most suitable formulations are likely to be dispersible tablets or multiparticulate formulations as these offer the benefits of flexible dosing with a suitable shelf-life, stability under Zone III and IV climate conditions and a simple manufacturing process not requiring specialised expertise or equipment.

## Data Availability

Not applicable.

## Abbreviations

API: Active pharmaceutical ingredient
BPCA: Best Pharmaceuticals for Children Act
DNDi: Drugs for Neglected Diseases Initiative
EDCTP: European & Developing Countries Clinical Trials Partnership
EMA: European Medicines Agency
EML: WHO essential medicines list
EMLc: WHO essential medicines list for children
EPAR: European public assessment reports
EU: European Union
EU-PR: European Union Paediatric Regulation
FDA: Food and Drug Administration
FDASIA: Food and Drug Administration Safety and Innovation Act
HINARI: Health Inter-network Access to Research Initiative
HIV: Human immunodeficiency virus
ICH: International Council for Harmonisation of Technical Requirements for Pharmaceuticals for Human Use
iPSP: Initial Paediatric Study Plan
LMIC: Low- and middle-income countries
MDGH: Medicines development for global health
NDA: New drug application
NTD: Neglected tropical disease
PCT: Preventive chemotherapy
PIL: Patient information leaflet
PK-PD: Pharmacokinetic-pharmacodynamic
PIP: Paediatric Investigation Plan
PREA: Pediatric Research Equity Act
PUMA: Paediatric use marketing authorisation
SPC: Summary of product characteristics
SRA: Stringent regulatory authority
STH: Soil transmitted helminths
US FDA: United States Food and Drug Administration
WHA: World Health Assembly
WHO: World Health Organization
WHOPAR: World Health Organization Public Assessment Report
WHO/TDR: UNICER/UNDP/World Bank/WHO Special Programme for Research and Training in Tropical Diseases
